# Cavitated Charcoal—An Innovative Method for Affecting the Biochemical Properties of Soil

**DOI:** 10.3390/ma14092466

**Published:** 2021-05-10

**Authors:** Krzysztof Gondek, Monika Mierzwa-Hersztek, Wojciech Grzymała, Tomasz Głąb, Tomasz Bajda

**Affiliations:** 1 Department of Agricultural and Environmental Chemistry, Faculty of Agriculture and Economics, University of Agriculture in Krakow, Al. Mickiewicza 21, 31-120 Krakow, Poland; monika6_mierzwa@wp.pl; 2 Faculty of Geology, Geophysics and Environmental Protection, AGH University of Science and Technology, Al. Mickiewicza 30, 30-059 Krakow, Poland; 3 BIRKO PROJEKT, Ul. Żabia 4/23, 05-220 Zielonka, Poland; birkoprojekt@gmail.com; 4 Department of Machinery Exploitation, Ergonomics and Production Processes, Faculty of Production and Power Engineering, University of Agriculture in Krakow, Ul. Balicka 116B, 31-149 Krakow, Poland

**Keywords:** cavitation, charcoal, enzymatic activity, heavy metals, plant, soil

## Abstract

Thermal biomass transformation products are considered to be one of the best materials for improving soil properties. The aim of the study was to assess the effect of charcoal after cavitation on the chemical and biochemical properties of soil. The study was carried out with a 10% aqueous charcoal mixture that was introduced into loamy sand and clay at rates of 1.76%, 3.5%, 7.0%, and 14.0%. The effect of the application of cavitated charcoal was tested on *Sorghum saccharatum* (L.). Soil and plant material was collected to determine chemical and biochemical properties. The application of cavitated charcoal reduced the acidification of both soils. The highest rate (14.0%) of cavitated charcoal increased the content of soil total carbon (C_Tot_) by 197% in the loamy sand compared to C_Tot_ in the control treatments, 19% for clay soil, respectively. The application of cavitated charcoal did not significantly change the total content of heavy metals. Regardless of the element and the soil used, the application of cavitated charcoal reduced the content of the CaCl_2_-extracted forms of heavy metals. Following the application of cavitated charcoal, the loamy sand soil presented an even lower content of the most mobile forms of the studied elements. It should also be noted that regardless of the soil texture, mobile forms of the elements decreased with the increased cavitated charcoal rate. The results of dehydrogenase and urease activity indicated the low metabolic activity of the microbial population in the soils, especially with the relatively high rates (7.0% and 14.0%) of cavitated charcoal. However, the cavitated charcoal used in the study showed a significant, positive effect on the amount of biomass *S. saccharatum* (L.), and its application significantly reduced the heavy metal content in the biomass of *S. saccharatum* (L.).

## 1. Introduction

The appropriate use of waste biomass is one of the ways to achieve sustainable agriculture in the 21st century. This approach will help create a product capable of improving soil characteristics in terms of its chemical and biological properties, as well as the quantity and chemistry of obtained plant biomass [1]. Significant amounts of waste biomass are generated in the world, and the instability of this waste makes its transformation often a key problem for the environment. The product of thermal biomass transformation is charcoal with properties similar to biochar. Solid products of thermal biomass transformation, due to their multiple specific properties, are often referred to as environmentally friendly materials [2,3,4]. The beneficial effects of these materials on soil water retention capacity, cation exchange capacity, and improvement of soil function as a carbon reservoir are usually observed because of the high C content and their resistance to microbial degradation [5,6,7].

Charcoal presents positive effects on soil cation exchange capacity what reflects in concentrations of available Ca and Mg and increases in soil pH [8]. Charcoal incorporation also decreases nitrate flux in soil leachate from mineral soils [9]. Cui et al. [10] reported that leachability, availability and bioaccessibility of Cu and Cd decreased in charcoal amended soils. Charcoal has a positive effect on the decomposition processes of soil organic matter [11]. However, sometimes negative effects of charcoal on decomposition are reported [12]. Charcoal incorporation influenced native soil organic matter content but this effect depends on soil type, charcoal type and time scale [13].

Despite many studies on the effect of charcoal and biochar on the environment, it was not possible to clearly define the mechanisms of their action [14]. The increased number of tests carried out have proven that the effect of thermally transformed organic materials on soil and plant properties is varied. Their effect is also conditioned by, among others, the type of feedstock, production conditions, material rate, location of tests and the type of plant cultivated [2,15,16]. The high porosity of charcoal associated with a broad range of pore sizes results in the capability to adsorb molecules and bacteria. Because of the composition of charcoal, adsorption is presumably governed by the van der Waals forces. Thus, the adsorption capacity of charcoal can be improved by higher surface area, heterogenous pore size and the shape of the particles [17,18].

Deenik et al. [2] demonstrated that the independent application of thermally transformed organic materials worsened the conditions for plant growth and development, probably due to limited nitrogen access and stimulation of soil microorganismal growth. The study by Kloss et al. [19] indicated a similar plant response, especially in the first two years after the application of biochar. In addition, these authors noted a decrease in the content of some trace elements in plant biomass. According to Kloss et al. [19], even with additional mineral fertilisation, the use of thermally transformed organic materials creates the risk of a short-term reduction in plant growth and development. On the other hand, Steiner et al. [20] reported that charcoal significantly improved plant growth and productivity of rice (*Oryza sativa* L.) and sorghum (*Sorghum bicolor* L.) if fertilized with NPK.

Previous studies on charcoal and biochar were focused not only on determining the structure and chemistry of these materials but also on searching for new solutions aimed at enriching them with various components and modifying their production process [21,22,23]. Given the properties of charcoal and the way it is applied, as well as the resulting environmental problems, there is a need to identify alternative production methods (form) or preparations for use [24]. Due to the nature of the cavitation process, it is possible to homogenise cavitated materials with the implosion of gas bubbles. In the case of hydrodynamic and acoustic cavitation, cavitation bubbles are present in liquid as a result of local ruptures of the continuous medium caused by high tensile forces. These forces arise from local sudden pressure decreases that may occur either in hydrodynamic processes or in a high-intensity ultrasonic field (20 kHz–1 MHz) [25]. The cavitation process can be modified by adding various types of chemical substances or by changing the physical parameters of the process [26]. At this moment there is no study on cavitied charcoal used in plant production. It is particularly interesting how cavitated charcoal affects plant and soil chemical properties, especially heavy metal remediation capabilities.

The aim of the study was to determine the effect of charcoal after cavitation on the chemical and biochemical properties of soil, as well as on the quantity and chemistry of *Sorghum saccharatum* (L.) biomass.

## 2. Materials and Methods

### 2.1. Cavitation of Charcoal

The study was carried out on charcoal obtained from an industrial installation (Birko Project, Zielonka, Poland) located in the Bieszczady Mountains (southeastern Poland). The production process was performed using deciduous wood. Before starting dry distillation, feedstock was ground and treated with drying gases at 160–180 °C. Then, the material was placed in a retort. After closing the retort, the residual water evaporates, and the thermal decomposition of the wood begins. It is initiated by hot gases of 250–300 °C, moving from the central part of the retort (annealing zone). The process is exothermic. The organic mass of wood is charred. The partially charred wood mass has a reduced volume; hence, it moves down the retort and enters the annealing zone. Here, at 500–550 °C, the final form of charcoal is formed [27].

The cavitation process was carried out in a 10% *w/w* aqueous charcoal mixture (particle size of 0–0.5 mm). The suspension was placed in a tank to which a 2.5 kW cavitation pump (Birko Project, Zielonka, Poland) was connected. After passing through the pump, the suspension was returned to the tank (closed circuit). The micronisation time for 150 dm^3^ of the suspension was 2 h, and the temperature increase during the cavitation process was from 40 °C (initial stage) to 60 °C (final stage).

### 2.2. Chemical Composition of Charcoal after Cavitation

To characterize the properties of the charcoal suspension after cavitation (CHAR-C), the following properties were determined in the material: electrical conductivity—conductometrically, pH—potentiometrically, and the content of N–NH_4_ and N–NO_3_—using ion-selective electrodes. The contents of macroelements (P, K, Ca, and Mg) and trace elements (Cu, Fe, Mn, and Zn) were determined after mineralising the sample in a mixture of concentrated HNO_3_ and HClO_4_ (3:2). The content of the elements was determined in the solution by inductively coupled plasma optical emission spectrometry (ICP-OES, Perkin Elmer Optima 7300 DV, Waltham, MA, USA) [28]. The material chemical composition is presented in Table 1.

### 2.3. Scanning Electron Microscopy, X-ray Diffractometry and Infrared Spectroscopy of Dry Residue Charcoal after Cavitation

The mineral composition of dry residue charcoal after cavitation was characterized by scanning electron microscopy, X-ray diffractometry and infrared spectroscopy. Scanning electron microscopy observations were made using an FEI 200 Quanta FEG microscope with an EDS/EDAX spectrometer (Thermo Fisher Scientific, Waltham, MA, USA). XRD data were determined using a diffractometer Rigaku SmartLab (Rigaku, Tokyo, Japan) with a graphite monochromator and rotation Cu anode. The data acquisition was in the range of 5–75°2θ with a 0.05° gradation, and a counting time of 1 s per grade. Fourier-transform infrared (FTIR) spectra were determined using a Nicolet 7600 spectrometer (Thermo Fisher Scientific, Waltham, MA, USA) in the wave range of 4000 to 400 cm^−1^. Samples were prepared by the standard KBr method.

### 2.4. Growth Experiment

The experiment was carried out in 2019 on two soil types: loamy sand (LS) and clay (C) collected from the 0–0.2 m layer. These soil types are commonly found on arable land in southern Poland. The properties of the soils used in the experiment are presented in Table 2. Both types of soil were characterized by natural metal content according to standards of Regulation of the Minister for the Environment [29]. Selected soil types were used because of different texture and clay content (80 and 520 g kg^−1^), which has a decisive influence on chemical sorption properties.

The growth experiment was conducted in containers (volume of 600 cm^3^) with a capacity of 500 g of soil dry mass. The experiment was conducted in a completely randomized design with three replications. The experimental scheme was the same for LS and C soil types and included the following treatments: the control with no cavitated charcoal (LS-0; C-0), a treatment with a 1.76% addition of an aqueous solution of cavitated charcoal (LS-1; C-1), a treatment with a 3.5% (*v/w*) addition of an aqueous solution of cavitated charcoal (LS-2; C-2), a treatment with a 7.0% addition of an aqueous solution of cavitated charcoal (LS-3; C-3) and a treatment with a 14.0% addition of an aqueous solution of cavitated charcoal (LS-4; C-4) (Table 3).

After introducing cavitated charcoal, distilled water (up to 45% water capacity) was added to the soils and thoroughly mixed with the material. Subsequently, soil with the charcoal and control soil were placed in PVC containers. After 24 h, seeds of *S. saccharatum* (L.) SOS 101,116 were sown. During plant growth, the water content in the soil was maintained at 60% of the water capacity. The pots were placed in a greenhouse at 25 ± 3 °C for 56 days. Then, plant aboveground biomass was collected, and plant roots were washed out with distilled water. The collected biomass was dried to a constant weight at 105 °C, and then, the amount of biomass was determined. To determine the chemical composition, the plant material was ashed in a chamber furnace at 450 °C for 12 h, and the residue was dissolved in diluted (1:2) (*v/v*) nitric acid. The contents of the trace elements were determined by inductively coupled plasma optical emission spectrometry (ICP-OES, Perkin Elmer Optima 7300 DV, Waltham, MA, USA) [28].

### 2.5. Biochemical Properties of Soil

Soil samples for chemical and biochemical analysis were collected after plant harvesting. Three soil samples weight of 20 g were collected from each pot. The soil biological activity measurements included enzymatic activity and soil respiration. Dehydrogenase activity (DhA) was determined by the method in Thalmann [30] using triphenyltetrazolium chloride (TTC) as the electron acceptor. Soil samples were incubated at 37 ± 2 °C for 24 h. The DhA was determined by colorimetry using a Backman DU 640 spectrophotometer (Beckman Coulter, Woonsocket, MA, USA) at a wavelength of 546 nm.

Urease activity (Ure) was measured by the Zantua and Bremner [31] method, with urea as a substrate, after an 18 h incubation at 37 °C. The enzymatic activity of ureases was determined by colorimetry using a Backman DU 640 spectrophotometer at a wavelength of 470 nm.

Basal respiration (BR) was determined according to the ISO 16072:2002 (International Organization for Standardization, 2002) method. Moist soil subsamples (20 g) were incubated at 20 ± 2 °C for 24 h. The emitted CO_2_ was absorbed in a solution of 0.05 M NaOH and precipitated as BaCO_3_ with the addition of a 0.5 M BaCl_2_ solution [32]. Substrate-inducted respiration (SIR) was determined by the measurement of CO_2_ production 6 h after glucose addition [33]. The respiratory-activation quotient (QR) was calculated by dividing the BR rate by the SIR rate (ISO 17155:2012) [34].

### 2.6. Chemical Properties of Soil

In 1 mm of dried, sieved soil samples, the following parameters were determined: pH—potentiometrically and electrical conductivity (EC)—conductometrically. The sum of alkaline cations (S) and hydrolytic acidity (Hh) were determined by using the Kappen method. The total carbon (C_Tot_), total nitrogen (N_Tot_) and total sulphur contents were determined with a CNS analyser Vario MAX Cube (Elementar, Langenselbold, Germany) [35]. Bioavailable trace elements were extracted from the soil for 2 h with a 0.01 mol dm^−3^ solution of CaCl_2_ (soil:solution = 1:10) [36]. The total macroelements and trace elements were measured after ashing the sample at a temperature of 550 °C for 12 h and mineralization in nitric and perchloric acids (3:2 *v*/*v*). The studied elements were determined by inductively coupled plasma optical emission spectrometry (ICP-OES, Perkin Elmer Optima 7300 DV, Waltham, MA, USA) [29].

### 2.7. Statistical Analysis

The experiment was carried out in a completely randomized design with three replicates. The statistical software Statistica v. 13.3 (StatSoft Inc., Tulsa, OK, USA) was used for the analysis of variance (Table S1 in Supplementary Materials). The normality of data distribution was checked using the Shapiro–Wilk test. The homogeneity of variance was checked by Levene’s test. The differences between means were calculated using a Duncan test with a *p* < 0.05 level of significance. Pearson’s correlation coefficient (*r*) was used to analyse the correlation between the CHAR-C rate and biochemical parameters and biomass productivity (Table S2). For data where significant *r* values, the linear regression models were determined and the coefficients of determination (*R*^2^) were calculated.

## 3. Results

### 3.1. Properties of Cavitated Charcoal and Soils

The cavitated charcoal (CHAR-C) used in the study had an alkaline pH and high electrical conductivity (EC). The content of macronutrients, e.g., calcium and potassium, varied in the studied material (Table 1). The content of heavy metals was lower than that observed in biochar produced from plant biomass [37]. The LS and C soil types used in the study had different granulometric compositions and chemical properties (Table 2). Both soils were characterised by the natural content of heavy metals examined in this study.

### 3.2. Physical Analysis of Dry Residue CHAR-C

**Figure 1:**
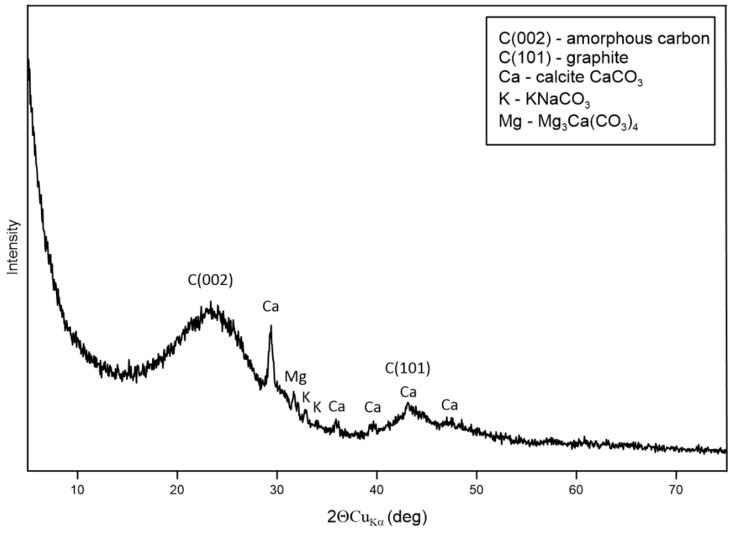
The X-ray diffraction pattern of dry residue cavitated charcoal.

**Figure 2:**
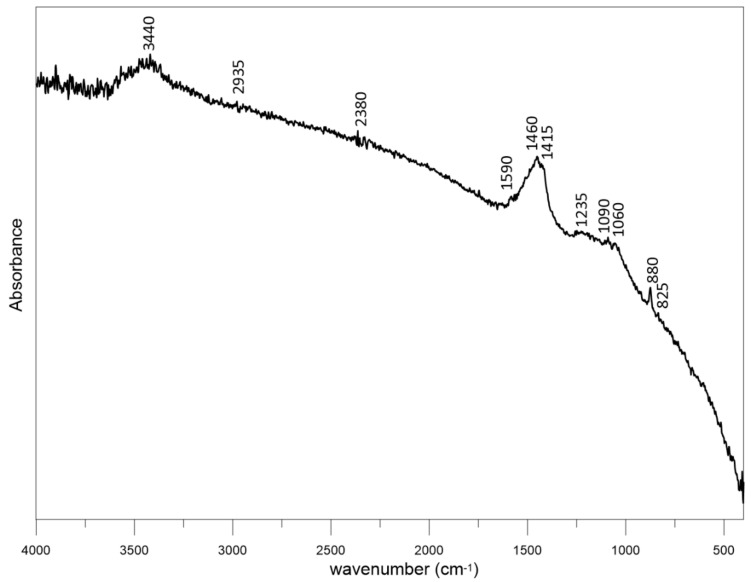
The FT-IR spectra of dry residue cavitated charcoal.

Figure 1 shows the X-ray diffraction pattern of dry residue cavitated charcoal. The wide range of C (002) diffraction peak (2θ = 15–30°) is the result of the amorphous structures of carbon. The weak and broad C (101) diffraction peak (2θ = 40–50°) can be ascribed to the a axis of the graphite structure. The mineral phases are represented by calcite (CaCO_3_) and trace amounts of potassium, sodium, and magnesium carbonates. Figure 2 shows the FT-IR spectra of cavitated charcoal. The presence of the band at 3440 is related to OH vibration. The weak peak at 2935 cm^−1^ is a result of the asymmetric C–H stretching vibration of CH_2_. Absorption at a wave number of 2380 cm^−1^ can be attributed to CO_2_ asymmetric stretching vibration. The peak observed at 1590 cm^−1^ is to the effect of C=C stretching at the aromatic C–C bond [38]. Two peaks centered at 1460 and 1415 cm^−1^ are due to the symmetric bending of CH_3_ [39]. The peaks around 1235, 1090, and 1060 cm^−1^ can be ascribed to C‒N stretching in aromatic tertiary amines [40]. The peak 880 cm^−1^ indicates C‒H out-of-plane bending in an aromatic ring. Finally, the band at 825 cm^−1^ is a result of N–H out of plane bending vibration.

**Figure 3:**
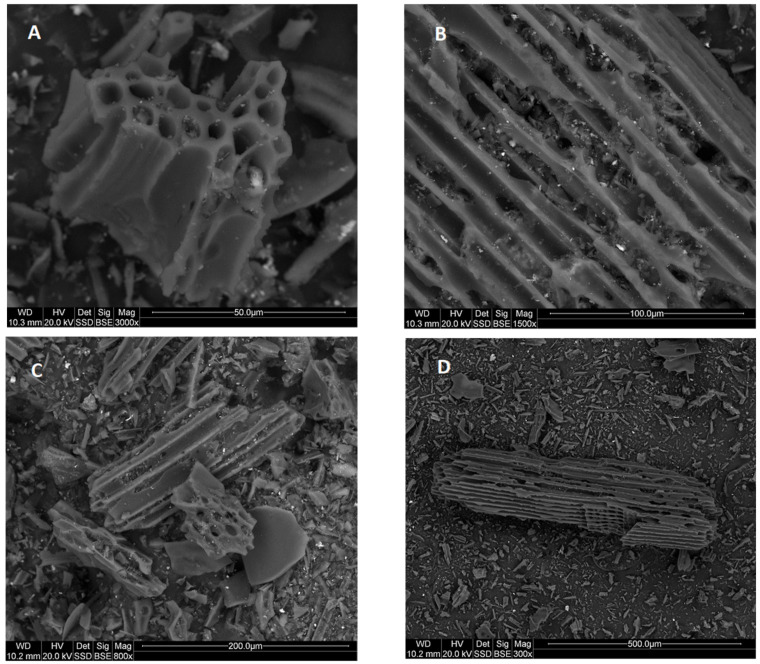
(**A**–**D**) The SEM of dry residue cavitated charcoal.

**Figure 4:**
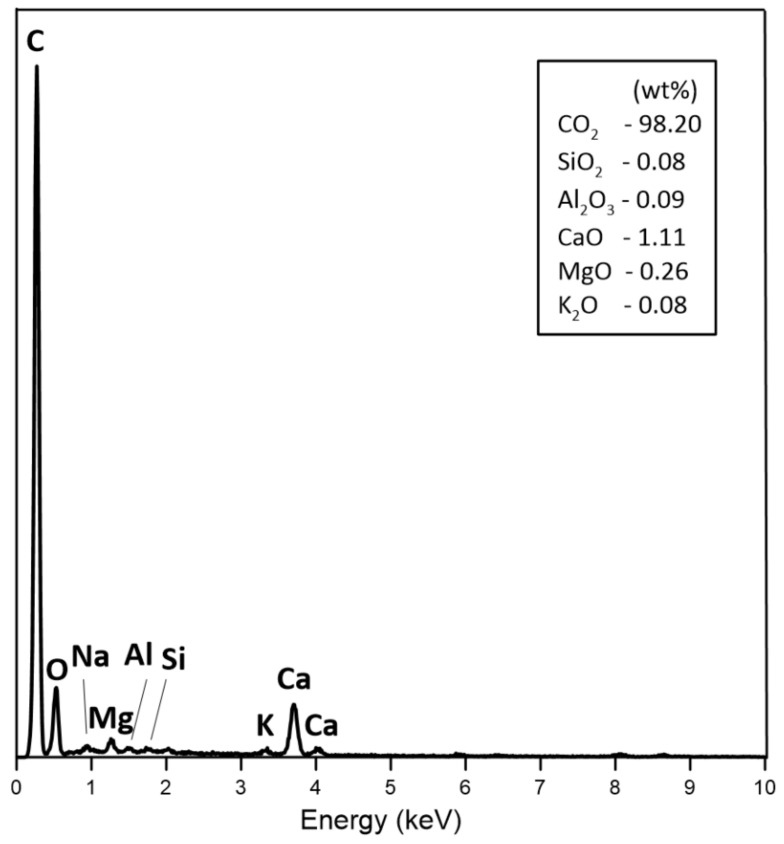
An analysis of the elemental composition of micro-samples of dry residue cavitated charcoal using EDS technique.

The SEM was used to present the morphology of the dry residue cavitated charcoal in Figure 3A–D. Figure 3A–D exhibits the general morphology of the sample, which is composed of porous particles in heterogenous sizes. The surface of the dry residues of cavitated charcoal was full of irregular cavities (Figure 3A,B), indicating that the porosity was developed by an activation agent. In the cavities, there are mineral grains composed of Ca, Mg, K, Na (Figure 4). Based on the XRD results we can conclude that there are calcium, magnesium, potassium carbonates (Figure 1, Figure 4).

### 3.3. Effect of the Addition of Cavitated Charcoal on Selected Chemical Properties of Soils

The application of CHAR-C reduced the acidification of the LS and C soil type (Table 4). Due to the greater buffering of the C soil than of the LS soil type, the efficiency of the deacidification effect of CHAR-C was lower. An increase in the electrical conductivity (EC) value was noted in proportion to the amount of CHAR-C introduced into the LS soil type. However, a significant increase in EC was observed only in the LS-4 treatment with the highest CHAR-C rate (Table 3). The introduction of the 7.0% (C-3) and 14.0% (C-4) rates of CHAR-C into the C soil significantly increased the EC values by 80% and 109%, respectively, compared to in the control soil (C-0). Due to the lack of data in the literature on the effect of cavitated charcoal on soil EC values, the obtained results were referenced in terms of the effect of applied biochar on the soil on the parameter value.

Significant differences in the total carbon (C_Tot_) content in the soils were found after using the same rates of CHAR-C (Table 3). Considering only the smallest rate of CHAR-C introduced into the LS soil type, an over 40% increase in the C_Tot_ content was noted, while this content in the C soil decreased by over 8% compared to that in the control. The highest rate of CHAR-C resulted in a 197% increase in the C_Tot_ content in LS, and a 19% increase in C compared to that in the control treatments. The total nitrogen content differed significantly but only between soils (Table 3). No significant changes in the total nitrogen (N_Tot_) content were noted after the application of CHAR-C.

### 3.4. Effect of the Addition of Cavitated Charcoal on the Content of Selected Heavy Metals in Soils

Significant differences in the studied element contents were dictated by the type of soil used (Table 5).

The effect of the application of CHAR-C on the bioavailability of heavy metals was tested after extracting the most mobile forms of Cu, Cd, Pb, and Zn with a 0.01 mol∙dm^−3^ solution of CaCl_2_ (Table 4). Regardless of the element and soil used, the application of CHAR-C reduced the content of CaCl_2_-extracted forms of heavy metals. Following the application of CHAR-C, the LS soil type presented a relatively low content of the most mobile forms of the studied elements. It should also be noted that, regardless of the soil, mobile forms decreased with an increased CHAR-C rate.

### 3.5. Effect of the Addition of Cavitated Charcoal on the Value of Selected Biochemical Parameters of Soils

**Figure 5:**
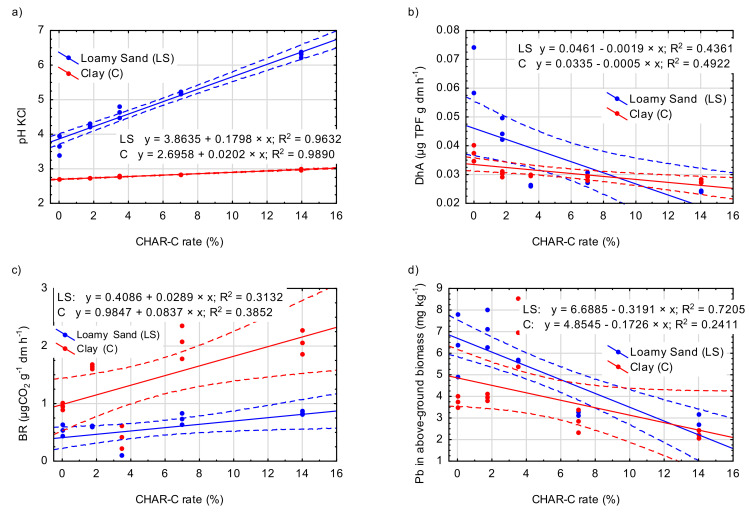
Relationship between the CHAR-C rate and chemical properties of soil: pH KCl (**a**), DhA (**b**), BR (**c**) and Pb in aboveground biomass (**d**). The solid line is the fitted linear regression, and the dotted lines represent the 0.95 confidence interval.

The values of basal respiration (BR) determined in our study differed significantly between the soils. The largest significant increase in the BR value compared to that in the control was found in treatments with 7.0% (LS-3 and C-3) and 14.0% (LS-4 and C-4) CHAR-C (Table 5). Compared to the control treatments, in LS-3 and LS-4, the BR increase was 38% and 58%, respectively, and in C-3 and C-4, it was 20% and 19%, respectively. The linear regression for the relationship between BR and the CHAR-C rate is presented in Figure 5.

The addition of glucose to the analysed soil samples significantly increased the respiration rate because of presence of easily available carbon in the microorganism habitat. Moreover, it means that there were no inhibitors in these soils, or their concentrations did not inhibit the viability of dormant microorganisms. Similar to BR, the largest increase in the SIR parameter was determined in soils with the highest CHAR-C rates. In both soils, the smallest rate of CHAR-C (LS-1, C-1) reduced the SIR value (Table 6).

The respiratory activity coefficient quotient (QR) determined in the study, which illustrates the number of dormant or active microorganisms, ranged from 0.11 to 0.14 for the LS soil and from 0.11 to 0.22 for the C soil (Table 5). The poorest microbiological parameters were found in soils without CHAR-C (LS-0, C-0) and with 8.8 mL (LS-1, C-1) and 17.5 mL (LS-2, C-2) of CHAR-C.

Table 5 presents the activity of dehydrogenases (DhA) associated with the transformation of carbon compounds and ureases (Ure) involved in the transformation of nitrogen compounds. The highest DhA and Ure activity occurred in the control treatments. Regardless of the soil and rate, the application of CHAR-C reduced the DhA activity (Figure 5). Compared to the control, the treatment with the highest rate of CHAR-C (70 mL) reduced the DhA activity by 57% in the LS soil type and by 24% in the C soil type.

The lowest CHAR-C rates (1.76% and 3.5%) significantly increased the Ure activity in all soils. The application of 7.0% and 14.0% rates drastically reduced the Ure activity in the LS soil, making the parameter value equal to that determined in the control soil. The Ure activity in the C soil type was also reduced under the influence of the same CHAR-C rates, but the decrease was not as rapid.

### 3.6. Effect of the Addition of Cavitated Charcoal on the Amount and Content of Selected Heavy metals in S. saccharatum (L.) Biomass

**Figure 6:**
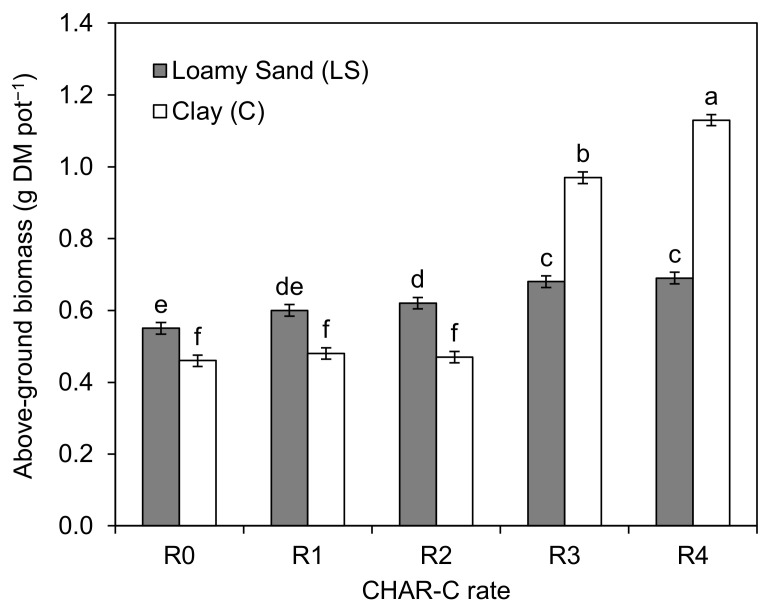
Dry matter of the aboveground biomass of *S. saccharatum* (L.). The mean values marked with the same letters do not differ significantly at *p* < 0.05 (according to Dancan’s test). R0, R1, R2, R3 and R4 correspond to 0.0 (control), 1.76, 3.5, 7.0 and 14.0% (*v/w*) rates of aqueous solution of cavitated charcoal, respectively.

**Figure 7:**
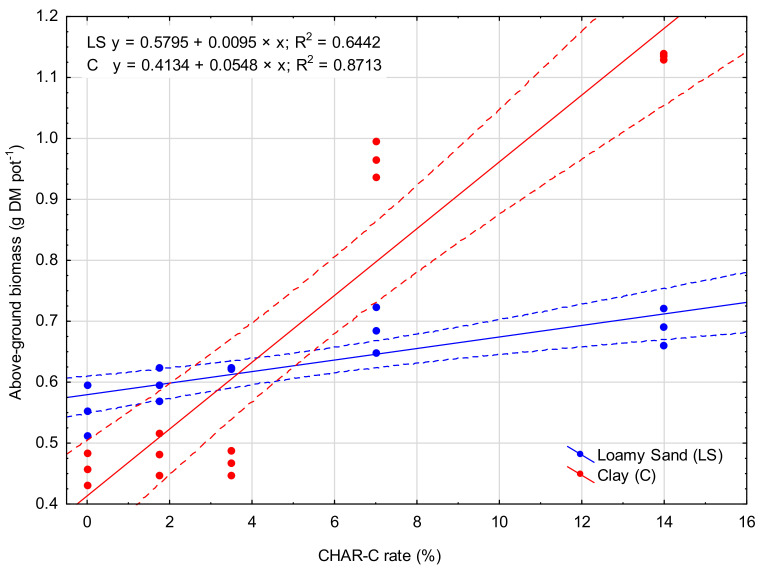
Relationship between the CHAR-C rate and aboveground biomass productivity of *S. saccharatum* (L.). The solid line is the fitted linear regression, and the dotted lines represent the 0.95 confidence interval.

Significantly higher amounts of *S. saccharatum* (L.) aboveground biomass compared to that in the control occurred in the LS soil type after applying 3.5% (LS-2), 7.0% (LS-3) and 14.0% (LS-4) rates of cavitated charcoal (Figure 6). For the C soil, the significant increase in the amount of *S. saccharatum* (L.) aboveground biomass occurred with the 7.0% (C-3) and 14.0% (C-4) rates. The largest amounts of biomass collected in the LS-4 and C-4 treatments were 25% and 145% higher, respectively, compared to that collected in the control treatments (LS-0 and C-0). The relationship between the aboveground biomass and CHAR-C rate is presented in Figure 7.

**Figure 8:**
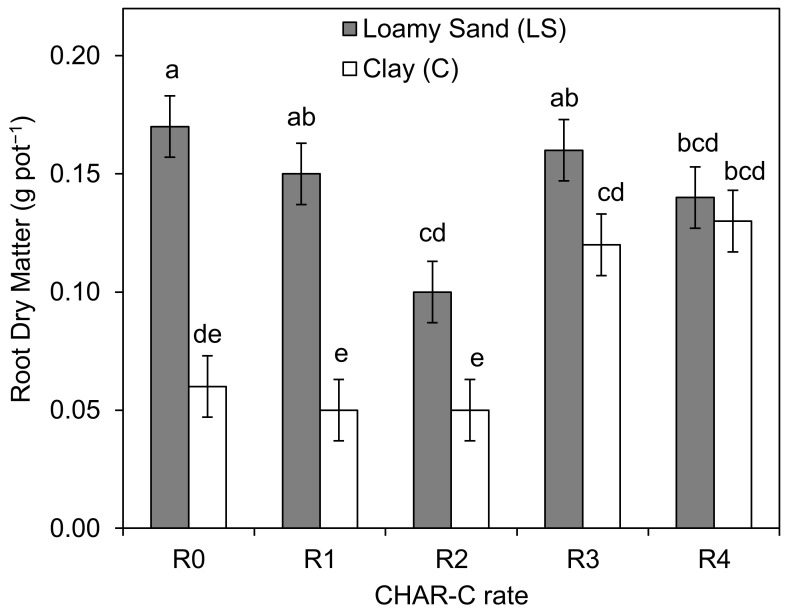
Root dry matter of *S. saccharatum* (L.). The mean values marked with the same letters do not differ significantly at *p* < 0.05 (according to Dancan’s test). R0, R1, R2, R3 and R4 correspond to 0.0 (control), 1.76, 3.5, 7.0 and 14.0% (*v/w*) rates of aqueous solution of cavitated charcoal, respectively.

The study revealed different trends in the amount of *S. saccharatum* (L.) root biomass in the LS and C soil types. For all CHAR-C rates, the amount of *S. saccharatum* (L.) root biomass was higher in the LS soil type than in the control (Figure 8). Higher rates of cavitated charcoal applied at C soil type (C-3 and C-4) resulted in significantly higher root dry matter of *S. saccharatum* (L.) than treatments with lower rates (C-1 and C-2).

The contents of the tested heavy metals in the aboveground biomass and roots of *S. saccharatum* (L.) differed mainly due to the soil type used (Table 7). The analysis of the contents of Cd, Pb, Zn and Cu in the aboveground biomass and roots of *S. saccharatum* (L.) revealed that the heavy metal content was lower with a higher CHAR-C rate in all soils (Figure 5). This trend was clearer for the aerial parts. However, it should be noted that the significantly higher contents of Cd, Zn and Cu in *S. saccharatum* (L.) biomass in the C soil type than in the other soils resulted from the greater acidification of that soil. The determined values of the tested heavy metals in *S. saccharatum* (L.) reflected changes in their availability in the soil.

## 4. Discussion

Due to the greater buffering of the clay soil than of the loamy sand soil, the efficiency of the deacidification effect of CHAR-C was lower. According to the literature, the application of charcoal in soil reduces soil acidification as a result of active compounds and alkaline elements accumulated in this material. The durability of the charcoal deacidifying effect depends on soil and climatic conditions and, as indicated by the published data, on the rate of alkaline cation leaching to the deeper layers of the soil profile [41,42].

The trend in the increased C_Tot_ in this study is confirmed by the reports on the charcoal effect on the carbon content in soil [42,43]. However, it should be noted that there are large discrepancies in the values of the increased C_Tot_ content in soils, which are probably dictated by the type of charcoal used, its rate, and climatic conditions [44]. Yang et al. [45] reported that biochar application at paddy fields in the rice–wheat rotation increased soil carbon content by 26%. Ma et al. [46] observed in a field experiment with wheat that biochar amendment can increase soil carbon content 2–4 times depending on rate and pyrolysis temperature. The beneficial effect of biochar application on organic carbon content is explained by (i) high carbon content in biochar particles and (ii) faster the formation and accumulation of soil organic matter [47,48]. Thus, biochar and charcoal are considered as new methods to capture and store carbon from the atmosphere [49].

There is a lack of information on the effect of the tested product on the N content in the soil. Hardy et al. [41] did not report significant differences in the nitrogen content between the soil fertilised with charcoal and the control soil. Hirsch et al. [50] discovered an even lower N content in soils into which charcoal was introduced. However, it should be noted that charcoal contains a nitrogen pool in a heterocyclic form that is practically inaccessible to plants, creating the need to include fertilisation with mineral forms of this element. Liao et al. [51] observed in a pot experiment with *Brassica napus* L. that pyrolyzed biomass did not affect soil nitrogen content. Similar results were observed by Kalu et al. [52] with pyrolyzed wood applied in a pot experiment with *Lolium multiflorum*.

Recent studies indicate that thermally transformed organic materials, including charcoal and biochar, exhibit sorption capacity for heavy metal ions [53,54]. These materials have a large specific surface area, i.e., a surface on which there are functional groups that are active in the sorbing of heavy metal ions. The sorption capacity of charcoal and biochar is determined, among others, by the concentration of heavy metals in the soil solution. In unpolluted soils, the natural consequence of using these materials is the reduction in the content of the most available element forms [53,54,55,56]. Gondek et al. [47], in a pot experiment with sandy soil, observed that thermally converted materials used as soil amendments reduced the mobility of heavy metals. This study confirms this effect, cavitated charcoal applied reduced the content of the most available heavy metals.

The respiratory activity of microorganisms in soil depends on their physiological state and environmental conditions. Although the large heterogeneity of the soil parameters, such as humidity, pH, temperature, availability of nutrients and structure, it is observed that soil respiratory activity is a very susceptible parameter closely linked to other soil biological properties [56,57]. As reported by Eisentraeger et al. [58], a respiratory activity coefficient ranging from 0.1 to 0.3 is low and points to a large amount of dormant microorganismal biomass as well as a low rate of bioremediation with potentially toxic substances in the soil. From this point of view, a high BR value is most beneficial for the soil. The poorest microbiological parameters were found in soils without CHAR-C (LS-0, C-0) and with 8.8 mL (LS-1, C-1) and 17.5 mL (LS-2, C-2) of CHAR-C. Similar results were also reported by Asirifi et al. [59] in a short-term study on sandy loam soil.

Biological processes affecting soil fertility and productivity are mainly associated with soil microbial activity, which translates into the number of enzymes produced [60]. Enzymatic activity is a very sensitive indicator of changes in soil after applying various materials. The literature presents both positive and negative effects of introducing thermally transformed organic materials into the soil [61,62,63,64]. The varied activity of individual enzymes can occur for many reasons. The most commonly indicated reasons in the literature are the content of C and N and the relationship between these components. Equally important is the available content of both elements [65]. One should also remember that the introduced rate of material is of key importance in maintaining optimal enzymatic activity in the soil. According to Asirifi et al. [59], thermally converted materials improve enzymatic activity in sandy loam soil under multi-crop rotation. Futa et al. [66] observed that dehydrogenase activity in loamy sand increased with increasing biochar rate.

This study showed that higher CHAR-C rates resulted in lower DhA, and Ure activity showed the low metabolic activity of the microbial population in the soils, especially at high rates (7.0% and 14.0%) of CHAR-C. The cause could have been, inter alia, increased EC values and limited availability of other nutrients. The study results published by Mierzwa-Hersztek et al. [67] indicated that the interaction of many factors significantly affects the enzymatic activity and microbiocenotic composition of the soil.

There are numerous reports in the literature on the significant fertilisation potential of thermally converted organic materials (charcoal, biochar). Some studies show that biochar and charcoal can improve plant growth in different ways. These materials can directly provide nutrients such as Ca, Mg, Al, Fe, P, S and K and indirectly amend the physical, chemical and biological properties of the soil [68]. Nutrient availability of nitrogen and phosphorus in the soil may be improved by adding pyrolyzed organic materials because of the higher cation adsorption and higher pH in acidic soils [22,69]. The increase in soil cation exchange capacity reduces nutrient leaching from the soil profile and increases nutrient availability to plants roots [70]. However, high sorption of biochar may immobilize plant nutrients, e.g., Ca, P, N, resulting in nutrient deficiency and further inhibiting plant growth [71]. According to Yao et al. [72], thermally converted organic materials can effectively absorb nitrate nitrogen, ammonium nitrogen in sandy soil. Zhang et al. [73] found that the application of pyrolyzed wheat straw at a low temperature strongly increases the availability of N, P and K in acidic soil with lower nutrient content. However, the properties of carbon materials, including pH, surface acid groups and ion exchange capacity, can have a great impact on the ability to absorb nutrients [54,56,74]. Our results indicate that in addition to improving soil properties, there is also a risk of limiting the availability of nutrients after applying charcoal or biochar to the soil.

In practice, it seems reasonable to combine charcoal or biochar applications with mineral fertilisers that meet the nutritional needs of plants, at least in the initial periods of their growth [2]. It is necessary to better understand not only sorption but also desorption of nutrients and metals because these are processes that, together with mineralization processes affect their concentration in soil solution. Consideration should be given to factors affecting the desorption of nutrients, such as soil types, feedstocks, conditions for thermal conversion and organic material rates.

## 5. Conclusions

Cavitated charcoal has great potential to deacidify soils and increase the C content. A higher deacidification effect is observed in loamy sand soil than clay soil because of the greater buffering capabilities of clay minerals. The cavitated charcoal used as soil amendment does not increase the total content of toxic metals; however, it substantially reduces their mobility. High rates of CHAR-C reduced toxic metal content in biomass of *S. saccharatum* (L.) in sandy loam and clay soils. This effect is more pronounced in clay soil because of the greater acidification of that soil. The determined values of the base respiratory activity of the soils into which CHAR-C was introduced were low, which indicates a large number of dormant microorganisms, as confirmed by the values of induced respiratory activity. The obtained results of DhA and Ure activity showed the low metabolic activity of the microbial population in the soils, especially at high rates (7.0% and 14.0%) of CHAR-C. The cavitated charcoal used in the study showed high-yielding properties, and its application significantly reduced the heavy metal content in the biomass of *S. saccharatum* (L.).

## Figures and Tables

**Table 1:** Selected chemical properties of cavitated charcoal (CHAR-C).

Parameter	Unit	Value
pH H_2_O	–	8.02
Electrical conductivity (EC)	mS cm^−1^	4.11
Dry matter	g dm^−3^	84.6
Organic matter	g dm^−3^	77.4
N–NH_4_	mg dm^−3^	56.0
N–NO_3_	mg dm^−3^	5.00
P	mg dm^−3^	247
K	mg dm^−3^	1214
Ca	mg dm^−3^	2343
Mg	mg dm^−3^	551
Na	mg dm^−3^	21.0
Cu	mg dm^−3^	3.52
Zn	mg dm^−3^	27.5
Cd	mg dm^−3^	0.302
Pb	mg dm^−3^	0.22

**Table 2:** Selected chemical and physical properties of the soil (± standard deviation).

Parameter	Unit	Loamy Sand (LS)	Clay (C)
pH H_2_O	–	5.90 ± 0.03	5.08 ± 0.04
pH KCl	–	4.55 ± 0.02	3.55 ± 0.06
Electrical conductivity (EC)	µS cm^−1^	24 ± 0	34 ± 3
Hydrolytic acidity (Hh)	mmol(+) kg^−1^	26.80 ± 3.44	130.95 ± 2.01
Sum of alkaline cation (S)	mmol(+) kg^−1^	51.38 ± 7.66	120.68 ± 18.38
Total Carbon	g kg^−1^	4.30 ± 0.87	26.56 ± 0.59
Total Nitrogen	g kg^−1^	0.38 ± 0.07	2.75 ± 0.03
Total Sulphur	g kg^−1^	2.32 ± 0.69	0.15 ± 0.01
Total Magnesium	g kg^−1^	0.28 ± 0.01	2.81 ± 0.02
Total Potassium	g kg^−1^	0.31 ± 0.03	3.34 ± 0.02
Total Phosphorus	g kg^−1^	0.16 ± 0.00	0.68 ± 0.00
Total Copper	mg kg^−1^	1.99 ± 0.16	9.67 ± 0.45
Total Cadmium	mg kg^−1^	0.45 ± 0.17	0.68 ± 0.04
Total Lead	mg kg^−1^	22.6 ± 0.91	33.0 ± 0.39
Total Zinc	mg kg^−1^	25.3 ± 0.5	117.4 ± 0.4
Sand	g kg^−1^	830	170
Silt	g kg^−1^	90	310
Clay	g kg^−1^	80	520

**Table 3:** Experimental design and treatments.

Treatments	Soil Types	Charcoal Rate (% *v/w*)
LS-0	Loamy sand	0
LS-1	Loamy sand	1.76
LS-2	Loamy sand	3.50
LS-3	Loamy sand	7.00
LS-4	Loamy sand	14.00
C-0	Clay	0
C-1	Clay	1.76
C-2	Clay	3.50
C-3	Clay	7.00
C-4	Clay	14.00

**Table 4:** Chemical properties of the soil after application of CHAR-C (± standard deviation).

Treatment		pH H_2_O	pH KCl	EC uS cm^−1^	C_Tot_ g kg^−1^	N_Tot_ g kg^−1^
Loamy Sand (LS)	LS-0	5.53 ± 0.03 ^e^	3.66 ± 0.02 ^d^	25 ± 1 ^b^	4.1 ± 0.4 ^c^	0.39 ± 0.03 ^a^
	LS-1	5.73 ± 0.03 ^d^	4.26 ± 0.03 ^d^	16 ± 0 ^b^	5.8 ± 0.5 ^b,c^	0.41 ± 0.05 ^a^
	LS-2	6.10 ± 0.04 ^c^	4.63 ± 0.03 ^c^	24 ± 2 ^b^	6.2 ± 0.8 ^b^	0.37 ± 0.04 ^a^
	LS-3	6.38 ± 0.06 ^b^	5.20 ± 0.05 ^b^	35 ± 4 ^a,b^	6.6 ± 0.6 ^b^	0.35 ± 0.04 ^a^
	LS-4	7.34 ± 0.05 ^a^	6.30 ± 0.06 ^a^	59 ± 3 ^a^	12.2 ± 0.7 ^a^	0.39 ± 0.06 ^a^
Clay (C)	C-0	3.60 ± 0.02 ^c^	2.69 ± 0.03 ^c^	148 ± 6 ^c^	24.4 ± 0.9 ^b^	2.74 ± 0.03 ^a^
	C-1	3.55 ± 0.02 ^c^	2.74 ± 0.03 ^c^	109 ± 5 ^d^	22.4 ± 0.9 ^b^	2.54 ± 0.06 ^b,c^
	C-2	3.55 ± 0.02 ^c^	2.78 ± 0.02 ^c^	148 ± 7 ^c^	22.6 ± 0.8 ^b^	2.47 ± 0.04 ^c^
	C-3	4.06 ± 0.03 ^b^	2.84 ± 0.03 ^a,b^	267 ± 12 ^b^	27.7 ± 0.7 ^a^	2.59 ± 0.07 ^b^
	C-4	4.41 ± 0.03 ^a^	2.98 ± 0.03 ^a^	310 ± 9 ^a^	29.0 ± 0.5 ^a^	2.48 ± 0.06 ^c^

The mean values marked with the same letters in superscript do not differ significantly at *p* < 0.05. EC—electrical conductivity, C_Tot_—total carbon, N_Tot_—total nitrogen content.

**Table 5:** The total content of heavy metals (mg kg^−1^) and heavy metals extracted with 0.01 mol dm^−3^ CaCl_2_ in the soil after application of CHAR-C (± standard deviation).

Treatment		Total Content				Extracted with 0.01 mol dm^−3^ CaCl_2_			
		Cd_Tot_	Cu_Tot_	Pb_Tot_	Zn_Tot_	Cd	Cu	Pb	Zn
Loamy Sand (LS)	LS-0	0.25 ± 0.05 ^a,b^	2.20 ± 0.23 ^a^	23.1 ± 0.84 ^a^	24.7 ± 0.4 ^a^	0.05 ± 0.00 ^a^	nd	0.05 ± 0.00 ^a,b^	0.98 ± 0.04 ^a^
	LS-1	0.23 ± 0.05 ^c^	2.50 ± 0.15 ^a^	22.9 ± 0.93 ^a,b^	27.4 ± 0.5 ^a^	0.04 ± 0.00 ^a^	nd	0.04 ± 0.00 ^a,b^	0.80 ± 0.06 ^b^
	LS-2	0.28 ± 0.04 ^a^	2.44 ± 0.13 ^a^	23.2 ± 0.75 ^a^	26.6 ± 0.4 ^a^	0.03 ± 0.00 ^b^	nd	0.06 ± 0.00 ^a^	0.55 ± 0.07 ^b^
	LS-3	0.24 ± 0.05 ^a,b^	2.45 ± 0.26 ^a^	21.7 ± 0.87 ^b^	26.7 ± 0.4 ^a^	0.03 ± 0.00 ^b^	nd	0.04 ± 0.00 ^a,b^	0.34 ± 0.04 ^c^
	LS-4	0.25 ± 0.07 ^a,b^	2.65 ± 0.18 ^a^	22.9b ± 085 ^b^	27.2 ± 0.6 ^a^	0.01 ± 0.00 ^c^	nd	0.03 ± 0.00 ^b^	0.21 ± 0.05 ^c^
Clay (C)	C-0	0.75 ± 0.09 ^a^	10.2 ± 0.36 ^a^	31.8 ± 0.41 ^a^	109 ± 0.7 ^b^	0.32 ± 0.02 ^a^	0.06 ± 0.00^a^	0.10 ± 0.01 ^a^	5.64 ± 0.03 ^a^
	C-1	0.66 ± 0.08 ^b^	9.67 ± 0.46 ^a^	31.6 ± 0.38 ^a^	109 ± 0.6 ^b^	0.30 ± 0.03 ^b^	0.01 ± 0.00^a^	0.10 ± 0.01 ^a^	5.29 ± 0.04 ^a^
	C-2	0.66 ± 0.09 ^b^	10.1 ± 0.54 ^a^	30.9 ± 0.32 ^a^	109 ± 0.7 ^b^	0.30 ± 0.002 ^b^	nd	0.08 ± 0.02 ^a,b^	5.16 ± 0.04 ^a^
	C-3	0.79 ± 0.14 ^a^	10.3 ± 0.49 ^a^	30.7 ± 0.37 ^a^	112 ± 0.4 ^a^	0.27 ± 0.002 ^c^	nd	0.06 ± 0.01 ^b^	4.67 ± 0.05 ^b^
	C-4	0.68 ± 0.10 ^b^	10.0 ± 0.39 ^a^	30.5 ± 0.36 ^a^	111 ± 0.5 ^a,b^	0.23 ± 0.01 ^d^	nd	0.07 ± 0.01 ^b^	3.46 ± 0.06 ^c^

The mean values marked with the same letters in superscript do not differ significantly at *p* < 0.05.

**Table 6:** Biochemical indicators of the soil after the application of CHAR-C (± standard deviation).

Treatment		BR (µgCO_2_ g^−1^ dm h^−1^)	SIR (µgCO_2_ g^−1^ dm h^−1^)	QR Ratio	DhA (µg TPF g dm h^−1^)	Ure (mg NH_4_ kg^−1^ h^−1^)
Loamy Sand (LS)	LS-0	0.53 ± 0.06 ^c^	4.7 ± 0.6 ^c^	0.11 ± 0.01 ^c^	0.056 ± 0.009 ^a^	0.8 ± 0.0 ^c^
	LS-1	0.60 ± 0.09 ^b^	4.5 ± 0.6 ^c^	0.14 ± 0.02 ^a^	0.045 ± 0.010 ^a,b^	7.7 ± 0.2 ^a^
	LS-2	0.61 ± 0.06 ^b^	5.3 ± 0.8 ^b^	0.11 ± 0.01 ^c^	0.026 ± 0.008 ^c^	6.5 ± 0.6 ^b^
	LS-3	0.73 ± 0.07 ^a,b^	6.4 ± 0.7 ^a,b^	0.11 ± 0.01 ^c^	0.028 ± 0.009 ^c^	0.8 ± 0.1 ^c^
	LS-4	0.84 ± 0.08 ^a^	6.6 ± 0.9 ^a^	0.13 ± 0.03 ^b^	0.024 ± 0.06 ^c^	0.8 ± 0.1 ^c^
Clay (C)	C-0	0.94 ± 0.11 ^c^	8.3 ± 0.6 ^c^	0.11 ± 0.03 ^d^	0.037 ± 0.004 ^a^	3.7 ± 0.6 ^d^
	C-1	1.64 ± 0.15 ^b^	7.8 ± 0.7 ^c^	0.22 ± 0.02 ^a^	0.030 ± 0.008 ^b^	12.9 ± 0.4 ^a^
	C-2	1.41 ± 0.13 ^b^	10.6 ± 0.8 ^b^	0.13 ± 0.04 ^c^	0.030 ± 0.005 ^b^	11.9 ± 0.7 ^a^
	C-3	2.07 ± 0.14 ^a^	13.3 ± 0.8 ^a^	0.16 ± 0.03 ^b^	0.029 ± 0.006 ^c^	8.8 ± 0.6 ^c^
	C-4	2.06 ± 0.10 ^a^	14.8 ± 0.9 ^a^	0.14 ± 0.03 ^b^	0.028 ± 0.005 ^c^	10.1 ± 0.5 ^b^

The mean values marked with the same letters do not differ significantly at *p* < 0.05. BR—basal respiration, SIR—substrate-inducted respiration, QR—respiratory-activation quotient, DhA—dehydrogenase activity, Ure—urease activity.

**Table 7:** Heavy metals content (mg kg^−1^) in the aboveground parts and roots of *S. saccharatum* (L.).

Treatment		Above-Ground Biomass				Roots			
		Cd	Pb	Zn	Cu	Cd	Cu	Zn	Pb
Loamy Sand (LS)	LS-0	1.92 ± 0.32 ^d^	6.35 ± 0.21 ^c,d^	120 ± 0.9 ^a,b^	14.0 ± 0.6 ^a,b,c^	10.9 ± 0.5 ^b,c,d^	31.1 ± 0.8 ^c,d^	606 ± 32 ^b,c^	29.5 ± 0.7 ^a^
	LS-1	2.66 ± 0.42 ^d^	7.13 ± 0.32 ^d^	153 ± 1.3 ^b^	12.1 ± 0.9 ^a,b^	7.7 ± 0.5 ^b,c,d^	18.8 ± c1.2 ^d^	531 ± 21 ^c^	16.8 ± 0.6 ^a,b^
	LS-2	1.97 ± 0.43 ^d^	5.66 ± 0.45 ^c^	149 ± 1.6 ^a,b^	12.4 ± 0.4 ^a,b^	11.7 ± 0.8 ^b,c,d^	14.2 ± 0.7 ^c,d^	679 ± 26 ^b,c^	19.9 ± 0.5 ^a,b,c^
	LS-3	1.13 ± 0.39 ^e^	3.25 ± 0.26 ^a,b^	112 ± 0.8 ^a,b^	11.9 ± 0.8 ^a,b^	6.7 ± 0.04 ^c,d^	13.0 ± 0.5 ^d^	200 ± 28 ^d^	20.6 ± 0.6 ^a,b^
	LS-4	0.68 ± 0.29 ^e^	2.62 ± 0.39 ^a,b^	83 ± 1.3 ^a^	11.0 ± 0.7 ^a^	4.6 ± 0.7 ^d^	20.1 ± 0.4 ^c,d^	125 ± 16 ^d^	17.0 ± 0.8 ^b,c^
Clay (C)	C-0	10.4 ± 0.84 ^c^	3.75 ± 0.34 ^b^	360 ± 1.6 ^d^	14.8 ± 0.8 ^a,b,c^	9.8 ± 0.6 ^b,c,d^	35.3 ± 0.9 ^b,c,d^	91 ± 12 ^b,c^	19.7 ± 0.9 ^a,b,c^
	C-1	9.7 ± 0.95 ^c^	3.96 ± 0.42 ^b^	260 ± 2.1 ^c^	16.6 ± 0.7 ^a,b,c^	14.7 ± 0.4 ^b,c^	65.5 ± 0.8 ^a^	1060 ± 38 ^a^	24.9 ± 0.5 ^a,b^
	C-2	9.8 ± 0,75 ^c^	3.95 ± 0.048 ^b^	232 ± 1.5 ^c,d^	21.7 ± 0.6 ^d^	11.7 ± 0.7 ^a,b,c,d^	38.6 ± 0.9 ^b,c^	760 ± 25 ^b,c^	21.2 ± 0.6 ^a,b^
	C-3	11.4 ± 0.68 ^b^	2.83 ± 0.27 ^a,b^	285 ± 2.3 ^c^	18.7 ± 0.8 ^c,d^	6.7 ± 0.5 ^c,d^	32.4 ± 0.9 ^c,d^	807 ± 29 ^b^	9.4 ± 0.8 ^c^
	C-4	14.6 ± 0.91 ^a^	2.24 ± 0.36 ^a^	300 ± 2.9 ^c,d^	16.2 ± 0.8 ^b,c^	36.7 ± 0.6 ^a^	56.7 ± 0.7 ^a,b^	699 ± 26 ^b,c^	18.0 ± 0.09 ^b,c^

## Data Availability

Data available on request due to restrictions eg privacy or ethical.

## Supplementary Materials

The following are available online at https://www.mdpi.com/article/10.3390/ma14092466/s1, Table S1. Analysis of variance (two-way ANOVA), Table S2. Pearson’s correlation coefficients (*r*) for relationships between the CHAR-C rate and soil and plant characteristics.
