# Supplementary material for: Cavitated Charcoal—An Innovative Method for Affecting the Biochemical Properties of Soil
Source: Materials (Basel). 2021 May 10;14(9):2466. doi: 10.3390/ma14092466 (PMC8126090; doi:10.3390/ma14092466)
Supplement: Supplementary file 1 [file materials-14-02466-s001.zip › materials-1201933-supplementary.pdf]

materials

Article

Cavitated Charcoal—An Innovative Method for Affecting the Biochemical Properties of Soil

Krzysztof Gondek [1,*], Monika Mierzwa-Hersztek [1,2], Wojciech Grzymała [3], Tomasz Głąb [4,*] and Tomasz Bajda [2,*]

[1] Department of Agricultural and Environmental Chemistry, Faculty of Agriculture and Economics, University of Agriculture in Krakow, al. Mickiewicza 21, 31-120 Krakow, Poland
[2] Faculty of Geology, Geophysics and Environmental Protection, AGH University of Science and Technology, al. Mickiewicza 30, 30-059 Krakow, Poland
[3] BIRKO PROJEKT, ul. Żabia 4/23, 05-220 Zielonka, Poland; birkoprojekt@gmail.com
[4] Department of Machinery Exploitation, Ergonomics and Production Processes, Faculty of Production and Power Engineering, University of Agriculture in Krakow, ul. Balicka 116B, 31-149 Krakow, Poland
* Correspondence: rrgondek@cyf-kr.edu.pl (K.G.); rtglab@cyf-kr.edu.pl (T.G.); bajda@agh.edu.pl (T.B.)

Citation: Gondek, K.; Mierzwa-Hersztek, M.; Grzymała, W.; Głąb, T.; Bajda, T. Cavitated Charcoal—An Innovative Method for Affecting the Biochemical Properties of Soil. **2021**, *14*, 2466. https://doi.org/10.3390/xma14092466

Academic Editor: Tamas Varga

Received: 13 April 2021
Accepted: 4 May 2021
Published: date

Publisher's Note: MDPI stays neutral with regard to jurisdictional claims in published maps and institutional affiliations.

Copyright: © 2021 by the authors. Licensee MDPI, Basel, Switzerland. This article is an open access article distributed under the terms and conditions of the Creative Commons Attribution (CC BY) license (http://creativecommons.org/licenses/by/4.0/).

Table S1. Analysis of variance (two-way ANOVA).

Parameter	Soil		Rate		S × R	
	F	*p*	*F*	*p*	*F*	*p*
DM	48.38	<0.001	265.77	<0.001	138.10	<0.001
RDM	50.77	<0.001	7.28	0.001	3.88	0.017
pH H_2O	11,970.30	<0.001	489.35	<0.001	60.52	<0.001
pH KCl	2703.11	<0.001	167.46	<0.001	107.09	<0.001
EC	990.83	<0.001	77.35	<0.001	37.59	<0.001
Ntot	14,104.05	<0.001	8.01	0.001	7.13	0.001
Ctot	1908.44	<0.001	36.35	<0.001	6.00	0.002
BR	293.84	<0.001	77.98	<0.001	17.62	<0.001
SIR	195.56	<0.001	21.41	<0.001	5.84	0.003
DhA	4.65	0.043	10.59	<0.001	3.97	0.016
Ure	576.82	<0.001	131.32	<0.001	18.92	<0.001
QR ratio	18.57	<0.001	10.53	<0.001	4.68	0.008
Total content of heavy metals in soil						
Cd_{Tot}	3425.54	<0.001	10.24	<0.001	13.21	<0.001
Cr_{Tot}	11,267.16	<0.001	7.65	0.001	3.47	0.026
Cu_{Tot}	2036.76	<0.001	0.33	0.851	0.82	0.526
Fe_{Tot}	61,084.73	<0.001	16.76	<0.001	2.30	0.095
Mn_{Tot}	8347.27	<0.001	2.80	0.054	1.83	0.162
Ni_{Tot}	4848.49	<0.001	2.34	0.090	6.23	0.002
Pb_{Tot}	854.06	<0.001	2.64	0.064	0.96	0.452
Zn_{Tot}	21,996.03	<0.001	2.66	0.063	1.20	0.340
Heavy metals extracted with 0.01 mol·dm^{-3} $CaCl_2$						
Cd	26,960.08	<0.001	167.39	<0.001	41.70	<0.001
Cr	451.25	<0.001	7.38	0.001	4.16	0.013
Cu	898.36	<0.001	128.25	<0.001	53.86	<0.001
Fe	30.68	<0.001	6.44	0.002	3.34	0.030
Mn	37,899.64	<0.001	389.22	<0.001	209.45	<0.001
Ni	35,113.78	<0.001	225.73	<0.001	180.98	<0.001
Pb	84.49	<0.001	4.67	0.008	2.34	0.091
Zn	12,731.63	<0.001	180.35	<0.001	47.35	<0.001
Heavy metals in above-ground biomass						
Cd	3654.01	<0.001	15.12	<0.001	59.74	<0.001
Cr	6.72	0.017	5.88	0.003	3.62	0.023
Cu	27.67	<0.001	1.61	0.210	2.13	0.115
Fe	0.25	0.626	11.99	<0.001	2.78	0.055
Mn	3.66	0.070	11.54	<0.001	10.79	<0.001
Ni	134.49	<0.001	8.62	<0.001	8.29	<0.001
Pb	14.20	0.001	27.42	<0.001	8.23	<0.001
Zn	185.21	<0.001	2.19	0.107	2.89	0.049
Heavy metals in roots						
Cd	26.46	<0.001	3.75	0.020	9.88	<0.001
Cr	39.48	<0.001	5.60	0.003	1.62	0.209
Cu	20.90	<0.001	2.23	0.103	4.01	0.015
Fe	4.24	0.053	4.74	0.007	1.70	0.190
Mn	33.24	<0.001	6.72	0.001	4.08	0.014
Ni	17.15	0.001	3.22	0.034	1.76	0.177
Pb	1.02	0.324	2.41	0.083	3.04	0.041
Zn	61.77	<0.001	9.51	<0.001	8.67	<0.001

Table S2. Pearson's correlation coefficients (*r*) for relationships between the CHAR-C rate and soil and plant characteristics.

Parameter	CHAR-C Rate	
DM	0.748	*
RDM	0.279	
pH H_2O	0.371	*
pH KCl	0.415	*
EC	0.426	*
Ntot	−0.027	
Ctot	0.258	
BR	0.419	*
SIR	0.478	*
DhA	−0.558	*
Ure	−0.070	
QR ratio	−0.011	
Total content of heavy metals in soil		
Cd	−0.001	
Cr	−0.034	
Cu	0.017	
Fe	−0.031	
Mn	0.015	
Ni	−0.007	
Pb	−0.072	
Zn	0.017	
Heavy metals extracted with 0.01 mol·dm^{-3} $CaCl_2$		
Cd	−0.155	
Cr	−0.175	
Cu	−0.397	*
Fe	−0.048	
Mn	−0.190	
Ni	−0.156	
Pb	−0.333	
Zn	−0.229	
Heavy metals in above-ground biomass		
Cd	0.108	
Cr	−0.118	
Cu	−0.085	
Fe	−0.253	
Mn	−0.431	*
Ni	−0.342	
Pb	−0.648	*
Zn	−0.149	
Heavy metals in roots		
Cd	0.363	*
Cr	−0.116	
Cu	−0.033	
Fe	0.215	
Mn	−0.421	*
Ni	−0.099	
Pb	−0.345	
Zn	−0.388	*
